# An emergency room influx and trauma cases prediction in a Brazilian ophthalmological hospital by an ophthalmologist without code experience

**DOI:** 10.5935/0004-2749.2022-0130

**Published:** 2022-10-19

**Authors:** Luis Filipe Nakayama, Lucas Zago Ribeiro, Caio Vinicius Saito Regatieri

**Affiliations:** 1 Department of Ophthalmology, Escola Paulista de Medicina, Universidade Federal de São Paulo, São Pasulo, SP, Brazil

**Keywords:** Machine learning, Emergency services, hospital, Eye injuries, Models, statistical, Algorithms, Aprendizado de máquina, Serviço hospitalar de emergência, Traumatismos oculares, Modelos estatísticos, Algoritmos

## Abstract

**Purpose:**

The emergency medical service is a fundamental part of healthcare, albeit crowded emergency rooms lead to delayed and low-quality assistance in actual urgent cases. Machine-learning algorithms can provide a smart and effective estimation of emergency patients’ volume, which was previously restricted to artificial intelligence (AI) experts in coding and computer science but is now feasible by anyone without any coding experience through auto machine learning. This study aimed to create a machine-learning model designed by an ophthalmologist without any coding experience using AutoML to predict the influx in the emergency department and trauma cases.

**Methods:**

A dataset of 356,611 visits at *Hospital da Universidade Federal de São Paulo* from January 01, 2014 to December 31, 2019 was included in the model training, which included visits/day and the international classification disease code. The training and prediction were made with the Amazon Forecast by 2 ophthalmologists with no prior coding experience.

**Results:**

The forecast period predicted a mean emergency patient volume of 216.27/day in p90, 180.75/day in p50, and 140.35/day in p10, and a mean of 7.42 trauma cases/ day in p90, 3.99/day in p50, and 0.56/day in p10. In January of 2020, there were a total of 6,604 patient visits and a mean of 206.37 patients/day, which is 13.5% less than the p50 prediction. This period involved a total of 199 trauma cases and a mean of 6.21 cases/day, which is 55.77% more traumas than that by the p50 prediction.

**Conclusions:**

The development of models was previously restricted to data scientists’ experts in coding and computer science, but transfer learning autoML has enabled AI development by any person with no code experience mandatory. This study model showed a close value to the actual 2020 January visits, and the only factors that may have influenced the results between the two approaches are holidays and dataset size. This is the first study to apply AutoML in hospital visits forecast, showing a close prediction of the actual hospital influx.

## Abstract (pt)

**Objetivo:**

Esse estudo tem como objetivo criar um modelo de Machine Learning por um oftalmologista sem experiência em programação utilizando auto Machine Learning predizendo influxo de pacientes em serviço de emergência e casos de trauma.

**Métodos:**

Um dataset de 366,610 visitas em Hospital Universitário da Universidade Federal de São Paulo de 01 de janeiro de 2014 até 31 de dezembro de 2019 foi incluído no treinamento do modelo, incluindo visitas/dia e código internacional de doenças. O treinamento e predição foram realizados com o Amazon Forecast por dois oftalmologistas sem experiência com programação.

**Resultados:**

O período de previsão estimou um volume de 206,37 pacientes/dia em p90, 180,75 em p50, 140,35 em p10 e média de 7,42 casos de trauma/dia em p90, 3,99 em p50 e 0,56 em p10. Janeiro de 2020 teve um total de 6.604 pacientes e média de 206,37 pacientes/dia, 13,5% menos do que a predição em p50. O período teve um total de 199 casos de trauma e média de 6,21 casos/dia, 55,77% mais casos do que a predição em p50.

**Conclusão:**

O desenvolvimento de modelos era restrito a cientistas de dados com experiencia em programação, porém a transferência de ensino com a tecnologia de auto Machine Learning permite o desenvolvimento de algoritmos por qualquer pessoa sem experiencia em programação. Esse estudo mostra um modelo com valores preditos próximos ao que ocorreram em janeiro de 2020. Fatores que podem ter influenciados no resultado foram feriados e tamanho do banco de dados. Esse é o primeiro estudo que aplicada auto Machine Learning em predição de visitas hospitalares com resultados próximos aos que ocorreram.

## INTRODUCTION

The ophthalmological emergency department is a fundamental part of medical care, with the facility of immediate medical consultation in severe and urgent cases, such as ocular trauma, retinal detachment, and ocular infection^(1,2)^.

In several countries, there is a lack of investment in disease prevention, which has led to an increase in the demand for immediate treatment by a large portion of the population^(3)^ with crowded emergency rooms (ERs)^(1,4,5)^ and delayed and low-quality assistance to actual urgent cases. Nonurgent visits have been reported between 8% and 62% of the total visits, especially at self- referral service centers^(6)^.

A possible solution to ER overcrowding is a quicker and more efficient patient movement through the emergency department^(3)^. Machine-learning algorithms can provide a smart and effective estimation of emergency patient volume for staff planning^(7,8)^, but the development of predictive models in healthcare had, so far, been exclusive to artificial intelligence (AI) experts with coding and computer science knowledge.

Transfer learning, where previously designed models can be adapted for training a new task by companies such as Amazon, Google, and Microsoft, has democratized access to AI and enabled any individual to develop AI models^(9)^.

Past studies from Faes and Antaki proved the feasibility of automated machine learning (autoML) in ophthalmology models built by physicians without any coding experience^(9,10)^ for the prediction of patient sex through retinal fundus photos^(11)^, cardiovascular risk^(12)^, and visual acuity outcomes in neovascular age-related macular degeneration^(13)^.

This study aimed to create a machine-learning model designed by two ophthalmologists without any coding experience using the AutoML platform for predicting ophthalmological emergency department appointments and trauma cases.

## METHODS

This study included data from emergency department visits at the *Hospital da Universidade Federal de São Paulo*, an academic tertiary hospital in Brazil, from January 2014 to December 2019. This study was approved by the institutional review board and followed the Helsinki Tenants.

The data was obtained from the hospital database, which extracted information from the patient’s charts. The algorithm training was performed by 2 ophthal-mologists-LFN and LZR-with no previous coding and machine-learning experience after a period of self-study and reading the Amazon Web Services (AWS) Forecast documentation guide.

The outcome is a prediction of a monthly patient volume and trauma cases based on the ER’s previous 5 years’ visits and a comparison to the actual ER influx during the forecast period.

### Study cohort

We included all patients who were evaluated at the emergency department of Sao Paulo Hospital from Janua ry 01, 2014 to December 31, 2019. A total of 356,611 visits were included.

Every patient classified with the international classification of diseases (ICD) code of “S” - injuries - was included in the group of trauma patients.

### Dataset preparation and features collected.

The dataset was collected from relevant electronic medical records with nonidentified information. During the analysis, daily influx and trauma patients were included, classified according to the ICD classification, and imported to the Amazon Simple Storage Service.

In both the datasets, the columns were defined as item_id, timestamp, and target_value, according to the AWS Forecast guidelines, and the model was applied in predicting target_vaue according to item_id.

### Automated machine learning

The training and prediction were made with the Amazon Forecast (https://aws.amazon.com/pt/forecast/), a fully managed service that utilizes statistical and machine-learning algorithms to deliver highly accurate time-series forecasts.

In this forecast, the Amazon Quantile Regression Amazon Convolutional Neural Networks-a causal Con-volutional Neural Network-was applied.

The 5 years of visits to the ER were applied to predictor training.

### Validation process

The Amazon Forecast performs backtesting by splitting the dataset into the training and testing datasets and then providing the following metrics to evaluate the model: root mean square error, weighted quantile loss, mean absolute percentage error, mean absolute scaled error, and weighted absolute percentage error^(14)^.

In this model, we considered the average weighted quantile loss, weighted absolute percentage error, and root means square error for model evaluation.

Next, we compared the model forecasting with the actual patient’s income in January 2020.

### Statistical Analysis

Continuous variables were presented as the mean, median, range, and standard deviation. Categorical variables were presented as counts and percentages. We defined the statistical significance as p<0.05. All statistical tests and descriptions were performed using the JASP (JASP Team 2020-version 0.14.1).

## RESULTS

The study dataset included 356,611 emergency visits, with 2,191 days and a mean of 162.76 daily visits (SD 52.05), mean age of 40.55 years (SD 20.45 years), and included 7,027 trauma cases with a mean of 3.20 cases/day (standard deviation of 2.40). In 277,020 (77.68%) visits, the ICD code was filled in the medical records.

During the forecast period, the mean prediction rate of emergency patients was 216.27/day in p90, 180.75/day in p50, and 140.35/day in p10.

The accuracy metrics in daily volume prediction presented an average weighted quantile loss of 0.09, weighted absolute percentage error of 0.12, and root means a square error of 31.61.

During the forecast period, the mean predicted emergency patients were 7.42 trauma cases/day in p90, 3.99/day in p50, and 0.56/day in p10.

The accuracy metrics in daily volume prediction presented an average weighted quantile loss of 0.288, weighted absolute percentage error of 0.48, and root means a square error of 2.61.

**Figure 1:**
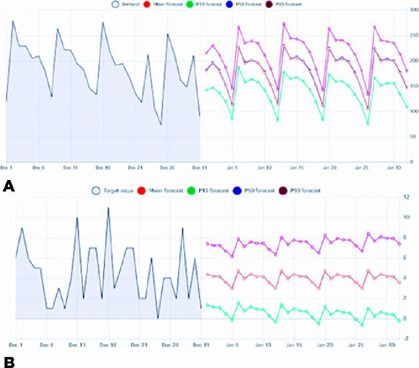
Cases of December 01 to 31, 2019, and prediction of January 01 to 30. (A) Daily emergency predicted cases. (B) Daily traumas predicted cases.

In the January 2020, there were a total of 6,604 patient visits and a mean of 206.37 patients/day, which is 13.5% less than that by the p50 prediction. This period included a total of 199 trauma cases and a mean of 6.21 cases/day, which is 55.77% more traumas than that by the p50 prediction. The daily distribution is illustrated in table 1 and the predictions in figure 1.

**Table 1:** Emergency room and trauma visits in January 2020 (forecast period)

Period	01/01	02/01	03/01	04/01	05/01	06/01	07/01	08/01	09/01	10/01	11/01	12/01	13/01	14/01	15/01	16/01	17/01	18/01	19/01	20/01	21/01	22/01	23/01	24/01	25/01	26/01	27/01	28/01	29/01	30/01	31/01	01/02
**Visits**																																
p10	141.8	146.6	136.9	120.6	85.6	186.2	157.5	163.5	158.2	142.6	119.8	83.1	177.5	164.2	167.1	159.7	143.3	118.0	83.9	173.2	159.1	159.7	150.4	133.4	112.2	75.6	165.9	152.0	155.4	155.6	133.7	109.0
p50	181.5	196.4	181.5	151.9	113.7	226.1	196.7	201.2	196.2	178.4	151.6	114.5	230.4	204.2	207.7	199.6	181.3	150.3	111.7	225.4	200.0	206.3	198.9	176.6	144.0	105.4	224.4	201.0	203.7	200.1	177.1	146.8
p90	215.4	229.7	212.5	186.9	145.6	266.4	234.7	238.8	234.6	210.8	183.9	143.6	273.1	244.5	243.2	237.0	217.1	182.9	142.1	263.6	241.3	240.5	232.3	212.6	180.5	134.0	267.3	240.7	237.9	234.5	213.1	180.2
Mean	71	226	211	204	123	302	249	233	246	240	166	107	275	228	230	225	183	183	119	307	255	210	205	185	146	132	287	260	215	209	209	163
**Trauma**																																
p10	1.3	1.1	1.1	0.5	−0.1	1.5	0.8	1.1	0.9	0.9	0.3	−0.3	1.3	0.6	1.0	0.8	0.7	0.1	-0.5	1.2	0.4	0.8	0.6	0.5	−0.1	−0.7	1.0	0.2	0.6	0.4	0.4	−0.2
p50	4.4	4.2	4.2	3.6	3.0	4.7	3.9	4.4	4.2	4.2	3.6	3.0	4.7	3.9	4.4	4.2	4.2	3.6	3.0	4.7	3.9	4.4	4.2	4.2	3.6	3.0	4.7	3.9	4.4	4.2	4.2	3.6
p90	7.4	7.2	7.2	6.7	6.1	7.9	7.1	7.6	7.4	7.4	6.9	6.3	8.0	7.3	7.8	7.6	7.6	7.0	6.5	8.2	7.5	7.9	7.8	7.8	7.2	6.7	8.4	7.7	8.1	8.0	7.9	7.4
Trauma	3	8	3	6	3	12	10	6	4	7	8	5	11	6	11	5	8	8	4	4	7	3	2	4	4	9	5	5	8	7	8	5

## DISCUSSION

The emergency medical department is a fundamental part of health care, but crowded ERs led to delayed and low-quality assistance in actual urgent cases^(1,4,5)^.

AI algorithms can provide intelligent and effective forecasting of emergency department influx to better plan the allocation of staff and hospital resources^(7,8)^. The development of AI models was previously restricted to data scientists who are experts in coding and computer science, but the transfer learning autoML has enabled AI development by any person without any coding experience.

Auto machine-learning modeling in image analysis is accessible to everyone with no coding experience, with feasibility proven by previous studies^(10)^.

This is the first study to apply an autoML model for the prediction of the ER influx and traumas cases with values close to the actual values recorded in January 2020 visits and traumas cases. This is a reproducible study that enabled an AI forecast prediction by a physician with no coding experience.

This study has some limitations. We opted to perform prediction in a prepandemic period of COVID19 due to the sudden decrease in patient volume after the pandemic period. This event may have influenced the results of the holidays on January 1 (New year) and January 25 (Birthday of Sao Paulo), the period of school holidays, and the dataset size, with a small prevalence of ocular traumas.

Incomplete ICD in medical records remains a problem in data quality, and we hence did not include these patients in trauma analysis.

More studies are warranted to assess the performance of auto ML using a more extensive dataset as well as to attempt to forecast patient visits in distinct daytimes for better staff planning.

This forecast applied a single AutoML platform to conduct a comparative study between other platforms such as Google’s and Microsoft Azure.

In conclusion, this is the first study to apply AutoML in hospital visits’ forecast prediction by 2 ophthalmologists with no prior coding experience to give a prediction close to that actual hospital influx.
